# How applicable is geospatial analysis in maternal and neonatal health in sub-Saharan Africa? A systematic review

**DOI:** 10.7189/jogh.12.04066

**Published:** 2022-08-09

**Authors:** Sisay Mulugeta Alemu, Abera Kenay Tura, Gabriel S Gurgel do Amaral, Catherine Moughalian, Gerd Weitkamp, Jelle Stekelenburg, Regien Biesma

**Affiliations:** 1 Global Health Unit, Department of Health Sciences, University Medical Center Groningen, Groningen, the Netherlands; 2 School of Nursing and Midwifery, College of Health and Medical Sciences, Haramaya University, Harar, Ethiopia; 3 Department of Obstetrics and Gynaecology, University Medical Centre Groningen, University of Groningen, Groningen, the Netherlands; 4 Department of Health Sciences, Community and Occupational Medicine, University of Groningen, University Medical Center Groningen, Groningen, the Netherlands; 5 Department of Cultural Geography, Faculty of Spatial Sciences, University of Groningen, Groningen, the Netherlands; 6 Department Obstetrics & Gynaecology, Leeuwarden Medical Centre, Leeuwarden, the Netherlands

## Abstract

**Background:**

Sub-Saharan Africa (SSA) has the world's highest maternal and neonatal morbidity and mortality and has shown the slowest progress in reducing them. In addition, there is substantial inequality in terms of maternal and neonatal morbidity and mortality in the region. Geospatial studies can help prioritize scarce resources by pinpointing priority areas for implementation. This systematic review was conducted to explore the application of geospatial analysis to maternal and neonatal morbidity and mortality in SSA.

**Methods:**

A systematic search of PubMed, SCOPUS, EMBASE, and Web of Science databases was performed. All observational and qualitative studies that reported on maternal or neonatal health outcomes were included if they used a spatial analysis technique and were conducted in a SSA country. After removing duplicates, two reviewers independently reviewed each study's abstract and full text for inclusion. Furthermore, the quality of the studies was assessed using the Joanna Briggs Institute (JBI) critical appraisal checklists. Finally, due to the heterogeneity of studies, narrative synthesis was used to summarize the main findings, and Preferred Reporting Items for Systematic Reviews and Meta-Analyses (PRISMA) guideline was strictly followed to report the review results. A total of 56 studies were included in the review.

**Results:**

We found that geospatial analysis was used to identify inequalities in maternal and neonatal morbidity, mortality, and health care utilization and to identify gaps in the availability and geographic accessibility of maternal health facilities. In addition, we identified a few studies that used geospatial analysis for modelling intervention areas. We also detected challenges and shortcomings, such as unrealistic assumptions used by geospatial models and a shortage of reliable, up-to-date, small-scale georeferenced data.

**Conclusions:**

The use of geospatial analysis for maternal and neonatal health in SSA is still limited, and more detailed spatial data are required to exploit the potential of geospatial technologies fully.

Sub-Saharan Africa (SSA) records the highest maternal and neonatal morbidity and mortality in the world [1]. Although SSA is home to only 13% of the world's population, 66% of maternal deaths worldwide occur in this region [2,3], with substantial inequality between and within countries in the region [3,4]. In addition, the region has shown the slowest progress in reducing maternal and neonatal morbidity and mortality [5].

Despite the global consensus to reduce maternal and neonatal morbidity and mortality [6,7], and despite these deaths being largely preventable [8], there are many challenges in SSA that slow progress. Low coverage, poor quality, and inequities in the provision of emergency obstetric and newborn care (EmONC) remain a challenge in many SSA countries [9]. In low- and middle-income countries (LMICs), and in particular, in the region of SSA, prioritizing interventions and resource allocation to areas where maternal and neonatal morbidity and mortality are most likely to happen, is necessary to ensure that the available resources are optimally used [10].

Geospatial analysis is one of the tools for improving decision-making by identifying geographical inequality and priority areas for implementation [11]. With the growing availability of spatial and/or temporal data at higher resolutions [12], the geographical coverage of maternal and neonatal health care can now be monitored with increasing accuracy. Geospatial analysis allows one to identify geographical inequalities in maternal and neonatal health by locating high and low morbidity and mortality areas. It can also help to determine the possible reasons for such disparities [13]. As a result, findings from geospatial analysis can help target intervention programs to areas where maternal and neonatal deaths are most likely to occur. Geospatial analysis can also suggest where to locate resources, such as ambulances and maternity waiting homes, to ensure timely access to EmONC facilities [14]. Other applications include identifying gaps in health facilities' availability and geographic accessibility, demand analysis, pinpointing ideal or priority locations for building new hospitals, health care data mapping and communication, and health program evaluation. Despite numerous applications of geospatial analysis techniques, its application for maternal and newborn health planning and priority setting in SSA was particularly rare. However, it is quickly gaining recognition, and more research outputs are being published. Therefore, there is a need to review the application of geospatial analysis in SSA. The aim of this review is, therefore, to explore the application of geospatial analysis in maternal and neonatal morbidity and mortality in SSA.

## METHODS

### Protocol registration

The review protocol was registered in the prospective registry of systematic reviews (PROSPERO) with registration ID CRD42021224930. We reported the review following the Preferred Reporting Items for Systematic Reviews and Meta-Analyses (PRISMA) guideline [15] (Appendix S1 in the **Online Supplementary Document**).

### Data source and search strategy

A systematic search of relevant publications was made in PubMed, SCOPUS, EMBASE, and Web of Science from inception until January 05, 2021. A comprehensive search strategy was developed for PubMed and adapted for the rest of the databases in consultation with a medical information specialist. The core search terms and phrases were 'maternal health,' 'neonatal health,' 'spatial analysis,' and 'sub-Saharan Africa'. The last search was done on January 05, 2021. In addition, bibliographies of identified articles and grey literature were hand-searched (Appendix S2 in the **Online Supplementary Document**).

### Eligibility criteria

All observational (ecological, cross-sectional, case-control, cohort, survey, and surveillance reports) and qualitative studies were included. In contrast, commentaries, case reports, case series, anonymous reports, conference abstracts, letters, protocols, systematic reviews, meta-analyses, and editorials were excluded. Articles were included if they fulfil the following criteria: 1) contain data on maternal or neonatal health outcomes (ie, morbidity, mortality, or health care utilization during pregnancy, childbirth, or postpartum), 2) used a spatial analysis technique, and 3) conducted in SSA countries. There was no restriction on language or time of publication of the study. However, studies that only describe the geographical distribution of health outcomes without spatial analysis (eg, comparing maternal mortality rates between different provinces of a country) were excluded. We also excluded one study because the full text was not accessible, and we failed to get a response from the corresponding authors.

### Screening and selection

**Figure 1:**
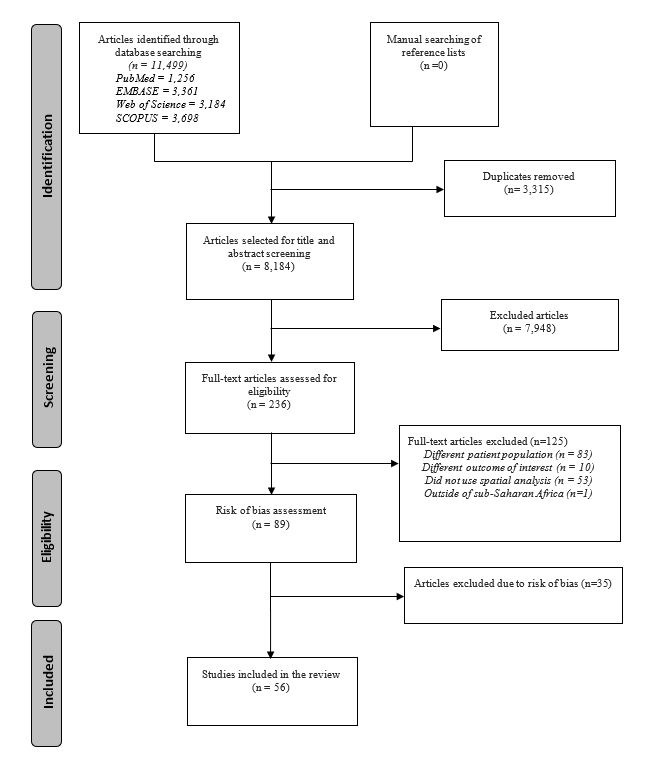
Preferred Reporting Items for Systematic Reviews and Meta-Analyses (PRISMA) flow diagram of literature screening and selection process.

Screening and selection of the studies were made using the Covidence, a web-based platform for systematic reviews [16]. At first, all studies obtained from the databases were exported to Covidence. After duplicates were removed, the title and abstract of each study were screened independently by two reviewers for inclusion. Then, two reviewers independently screened the full text of all potentially important articles. Any disagreement between reviewers was resolved by discussion. The agreement between reviewers during the screening was 'good' (Cohen's kappa = 0.63) [17]. The screening and selection process of the reviewed articles was illustrated using a PRISMA flow diagram (**Figure 1**).

### Quality assessment/ risk of bias and data extraction

The quality of studies that meet the eligibility criteria was assessed using the Joanna Briggs Institute (JBI) critical appraisal checklists. JBI comprises checklists that look at the methodological quality of a study to determine the extent to which it has addressed the possibility of bias in its design, conduct, and analysis [18]. Only studies with 'good' quality were selected for data extraction and analysis. For all included studies, data about the title, year of publication, journal of publication, aim or research questions, focus (ie, maternal, neonatal, or both), the scope of the study (regional, national, subnational), country, outcome variables, study design, data sources, study period, study participants/population, sample size, spatial analysis technique used, funding sources, findings, and the conclusion were extracted. For one-third of the studies, data extraction and quality assessment were done by two individuals independently. The agreement between the two individuals was assessed to be “good.” Subsequently, one reviewer did a quality assessment and extracted the remaining studies to save time.

### Data synthesis and reporting

We used narrative synthesis and a table to summarize the main findings. Meta-analysis was not possible since the included studies were heterogeneous in terms of population, methods, and outcomes. PRISMA guideline [15] was strictly followed to report the review results.

## RESULTS

### Characteristics of included studies

**Figure 2:**
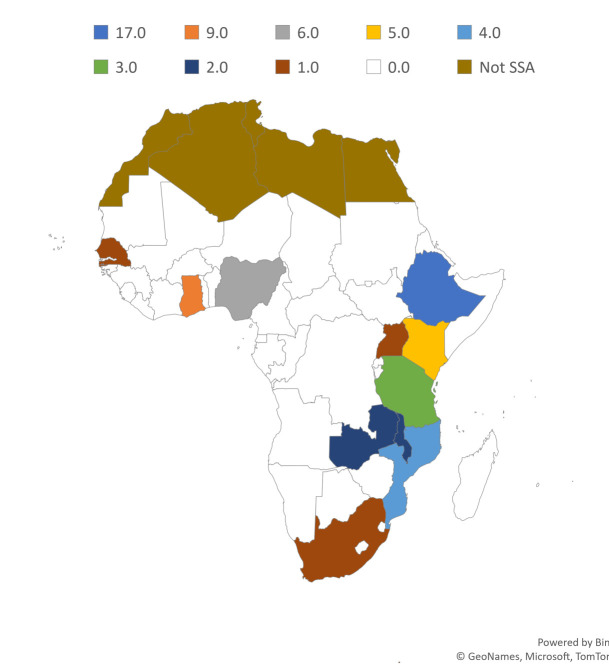
Number of national and subnational geospatial studies conducted on maternal and neonatal health outcomes in each sub-Saharan Africa (SSA) country.

From a total of 11 499 studies returned from the search, 56 studies that fulfilled the inclusion criteria were included in the narrative synthesis (**Figure 1**, Appendix S3 in the **Online Supplementary Document**). The majority (57%) of geospatial studies were national-level studies, while only 9% were regional studies. More than a quarter (30%) of the studies were done in Ethiopia. More than three-fourth (79%) of the studies were on maternal health, followed by neonatal health (12%) and both (9%). Nine in ten (91%) of the studies were cross-sectional, and a half (54%) used Demographic and Health Survey (DHS) data. Global spatial autocorrelation (29%), spatial scan statistics (27%), and different travel time/accessibility measuring techniques (23%) were the most commonly used spatial analysis methods (**Table 1** and **Figure 2**).

**Table 1:** Descriptive summary of the characteristics of geospatial studies in maternal and neonatal morbidity and mortality

		Number	Percent
**Study period**	Before 2000	1	1.8%
	2000 to 2009	9	16.1%
	2010 to 2014	22	39.3%
	2015 to 2021	24	42.9%
	Total	56	100%
**Scope**	National	32	57.1%
	Sub-national	19	33.9%
	Regional	5	8.9%
	Total	56	100%
**Focus**	Maternal	44	78.6%
	Neonatal	7	12.5%
	Both	5	8.9%
	Total	56	100%
**Country**	Ethiopia	17	30.4%
	Ghana	9	16.1%
	Nigeria	6	10.7%
	Kenya	5	8.9%
	Mozambique	4	7.1%
	Tanzania	3	5.4%
	Zambia	2	3.6%
	Malawi	2	3.6%
	Uganda	1	1.8%
	South Africa	1	1.8%
	Senegal	1	1.8%
	Regional *	5	8.9%
	Total	56	100%
**Outcome**	Skilled birth attendance, ANC, and PNC	32	57.1%
	Access to health care	12	21.4%
	Neonatal mortality	4	7.1%
	Pregnancy-related mortality	3	5.4%
	Perinatal mortality	2	3.6%
	Abortion	2	3.6%
	Stillbirth	1	1.8%
	Total	56	100%
**Study design**	Cross-sectional	51	91.1%
	Cohort	3	5.4%
	Case-control	1	1.8%
	Others	1	1.8%
	Total	56	100%
**Analysis method**	Global spatial autocorrelation	16	28.6%
	Spatial scan statistics	15	26.8%
	Travel time/accessibility modelling	13	23.2%
	Different logistic regression models	11	19.6%
	Hot spot analysis	10	17.9%
	Spatial interpolation	7	12.5%
	Different Bayesian models	7	12.5%
	Exploratory spatial analysis	6	10.7%
	Local spatial autocorrelation	3	5.4%
	Incremental spatial autocorrelation	2	3.6%
	Geographic Weighted Regression	2	3.6%
	Zero-inflated negative binomial model	2	3.6%
	Anselin Local Moran's I	2	3.6%
	Linear regression	2	3.6%
	Others	16	28.6%
	Total†	114	204%
**Data source**	DHS survey	30	53.6%
	Health facility surveys	10	17.9%
	Secondary analysis of RCT	4	7.1%
	Secondary analysis of cohort data	3	5.4%
	HDSS	3	5.4%
	HMIS	3	5.4%
	WorldPop database	3	5.4%
	Census	3	5.4%
	Primary data collection	3	5.4%
	Road network data	2	3.6%
	Others	13	23.2%
	Total‡	77	138%

ANC – antenatal care, PNC – postnatal care, DHS – demographic health survey, RCT – randomized control trial, HMIS – health management information systems, HDSS – health and demographic surveillance system

*Out of the five regional studies, one study in East (ie, Ethiopia, Kenya, Malawi, Mozambique, Tanzania, Zambia, and Zimbabwe) and West Africa (Benin, Burkina Faso, Ghana, Guinea, Mali, Niger, and Nigeria), one study contained 49 and another 48 countries, and the remaining two studies in Mali, Guinea and Liberia, Benin, Niger, and Nigeria.

^†^The total percentage is more than 100 because some studies used more than one analysis technique.

^‡^The total percentage is more than 100 because some studies used more than one data source.

### Application of geospatial techniques in Sub Saharan Africa

Geospatial analysis was applied for a) identifying inequalities in maternal and neonatal morbidity and mortality, and b) health care utilization; c) identifying gaps in availability and geographic accessibility of maternal health facilities; d) highlighting the effect of availability and accessibility on maternal health care utilization; and e) modelling intervention areas.

#### Identifying inequalities in maternal and neonatal morbidity and mortality

Three studies [19-21] applied geospatial analysis to describe the distribution of maternal and pregnancy-related mortality. First, a national study in Zambia [19] found significant differences in the country's lifetime risk of pregnancy-related deaths. This study showed a lower lifetime risk of pregnancy-related death clusters in Lusaka, Muchinga, Copperbelt, Northwestern, and Southern provinces. In contrast, clusters of higher lifetime risk were found in Western and Luapula provinces. Similarly, a study in two rural districts in southern Tanzania [20] showed geographical differences in maternal mortality even within a relatively small geographical area. Conversely, a study in a small geographic area on the west side of South Africa [21] with approximately 11 000 household inhabitants found no evidence of spatial clustering of maternal mortality in the study area.

Seven studies [13,22-27] reported the geographical distribution of stillbirth, perinatal, and neonatal mortality at regional, national, and subnational levels. A large study in all 49 SSA countries indicated no spatial clustering of neonatal mortality between countries [22]. On the other hand, a study in seven East African and seven West African countries found higher neonatal mortality in Ethiopia, Kenya, and Tanzania compared to the rest of the countries [23]. Studies in Malawi [24], Nigeria [25], and Ethiopia [26,27] also found inequalities in perinatal deaths within the countries. For instance, relatively lower perinatal deaths in Ethiopia were found in Addis Ababa, some parts of Tigray, Gambella, Benishangul-Gumuz, and Southern Nations Nationalities and Peoples Region, whereas significantly higher perinatal deaths were recorded in the Somali region, South Amhara, North Oromia, and Southern Afar [26,27]. For example, in one study, women within a specific hotspot area in the Somali region of Ethiopia had a 22.5 times higher risk of experiencing stillbirth than women outside the hotspot area [27].

Two studies [28,29] which were conducted in Ghana and Ethiopia on abortion, showed a significant variation in abortion rates in those countries [28,29]. In Ghana, there was a distinct north-south difference in the prevalence of abortion, with higher rates in the southern part of the country [28]. Similarly, in Ethiopia, significant spatial variation, where higher abortion was recorded in the northern Tigray region, border areas of Oromia, Amhara, and Southern Nations Nationalities and Peoples Region, was observed [29].

#### Identifying inequalities in maternal health care utilization

Twenty-seven studies investigated inequalities in maternal health care utilization [30-56]. All reported significant disparities in maternal health care utilization at regional, national, or subnational levels. For example, a regional study among five neighbouring West African countries showed that utilization was significantly higher in some regions of Nigeria and Benin, while the lowest utilization was reported among women residing in some areas of Niger and Mali [30].

Inequality in maternal health care utilization was also identified at a national level. For instance, in Ethiopia, a relatively higher utilization was found in Addis Ababa [31-37], Dire Dawa [31,34-37], Tigray [31,33-39], and Harari [31,33,35,36]. A relatively low utilization was found in regions of Somali [31,33,34,36,37,39], Afar [31,33-35,37], Oromia [32,33,35,36,39], most of Amhara [33-35,37,40], Gambela [32,33,37,39], Benishangul Gumuz [34,35], and parts of Southern Nations Nationalities and Peoples Region [32,34-36,39,40]. Similarly, a significant north-south divide in the likelihood of maternal health care utilization was reported in Nigeria. Most Northern states have a significantly lower maternal health services utilization compared with the southern states [41-45], except for a few southern states (such as part of Cross River [43], Bayelsa [42,43], Imo and Anambra state [45]). The north-south division in the utilization of maternal health care was also evident in Ghana, with women from the north being more likely to utilize maternal health care services [46-49]. However, one study in Ghana reported a significant difference between the southern and northern regions only for skilled birth attendance but not for antenatal care utilization [50]. A study in Uganda also reported marked geographic variation in maternal health care utilization across the country [51].

Most geospatial studies also demonstrated variation in maternal health care services utilization at a subnational level. For example, studies in Ethiopia [52,53], Kenya [54], and Mozambique [55] found subnational clustering of maternal health care utilization at the different administrative levels. Another study that focused on selected districts in Ethiopia's four most populous regions (Tigray, Oromia, Amhara, and Southern Nations Nationalities and Peoples Region) found evidence of geographic clustering in the utilization of skilled birth attendance but not for antenatal care [56].

#### Identifying gaps in availability and geographic accessibility of health facilities

A regional study from 48 countries [57] reported that all countries meet the geographical accessibility target of 80% of all women located within 2h of the nearest comprehensive emergency obstetric and newborn care (C-EmONC) at the national level except Eritrea (71%). This is with the assumption that all hospitals in SSA have a C-EmONC capacity. On the other hand, a national study in Ghana estimated only 55% of women in Ghana reach their nearest C-EmONC facility within two hours. Furthermore, 29% of Ghanaian women reside more than four hours from the C-EmONC facility [58]. However, substantial variations were also observed regarding geographic accessibility at the subnational level. The same study that reported all SSA countries meet the geographical accessibility target [57] also observed a substantial variability at subnational levels, with the majority (56%) of the countries having at least one administration unit not meeting the target at administrative level-1 (the largest subnational administrative unit of a country) and 74% at administrative level-2 (the largest subnational administrative unit of a country). Malawi, for example, has 82% of the country's administrative level-2 not meeting the target. Similarly, in Ethiopia, although the national average distance to EmONC facilities was 12.8 km, it varies from as long as 27.1 km in the Somali region to as short as a kilometre in bigger cities of Addis Ababa and Dire Dawa [32,38]. Similar phenomena were observed in Ghana, where 82% of women can access C-EmONC facilities within two hours in the metropolitan region of Greater Accra while only less than a quarter in the two predominantly rural Northern and Western regions [58]. Studies in Kenya, Malawi, Tanzania, Mali, Guinea, and Liberia also reported substantial disparities in access within the country and even at the subnational level [54,59-62].

Looking at the geographic accessibility during inter health facility referral, the median distance between health facilities that can(not) perform caesareans section was 32km (ie, 41-minute driving time) in Senegal [63]. On the contrary, in two regions of Ethiopia (Tigray and Amhara), the average time between the two facilities was as high as two hours [64].

Regarding availability, according to a regional study, all SSA countries meet the World Health Organization (WHO) availability of one C-EmONC facility per 500 000 population targets at the national level, except for Ethiopia (which only completes 77% of the target) and Senegal (96% of the target). In addition, there was an average of two hospitals per 500 000 people across SSA. However, at the subnational level, 58% of countries have at least one administrative unit not meeting this target at administrative level-1 (the largest subnational administrative unit of a country) and nearly all (95%) at administrative level-2 (the largest subnational administrative unit of a country) [57]. Also, in the Upper West region of Ghana, there was approximately one C-EmONC facility per 170 000 populations, thus exceeding the availability target by a factor of three. However, only one-third of women in that region can access these facilities within two hours. Similarly, in the Northern Region of Ghana, where one C-EmONC facility was present per 300 000 population, only a quarter can access a C-EmONC facility within two hours of travel [58].

#### Highlighting the effect of availability and geographic accessibility on maternal health care utilization

Most studies examining the effect of distance on health care utilization found that the further a woman lives from a health facility, the less likely she is to receive necessary maternal health care [38,53,65-67]. Some studies, however, found a threshold distance beyond which maternal health service utilization ceased to decline with additional distance [67,68]. In contrast, studies in Ghana and western Kenya did not find any correlation between distance to a health facility and maternal health care utilization [46,54].

Also, in two of the reviewed studies, the number of facilities around where a woman lives was strongly associated with maternal health care utilization [55,59]. Another study revealed that the effect of health service availability on maternal health care utilization was much stronger during childbirth than during pregnancy [65].

In addition to the availability and accessibility of health facilities, the quality of the service at the facility strongly influenced maternal health care utilization. Studies in different countries of SSA confirmed that higher service quality at health facilities increases the odds of maternal health service utilization [38,46,51]. However, two studies in different parts of Ghana failed to find such an association [65,68].

#### Modelling interventions

So far, only two studies [64,69] have used geospatial analysis to identify areas that can benefit most from specific interventions. Both studies, one of which is in Mozambique and the other in Ethiopia, modelled the impact of two interventions (ie, improving transportation and communication and upgrading strategically selected facilities) on the population's access to facilities.

In Mozambique, upgrading 37 geographically strategic health facilities to perform caesarean delivery would result in an additional 4% of the population (equivalent to 968 846 people) reaching a higher health facility within 2 hours. For the remaining population, access would be improved even though it would still take more than 2 hours to get to a higher health facility. Furthermore, when the above intervention was combined with making ambulances/transportation and communication in facilities available where none exist, it reduced mean inter-facility referral travel time from 2.5 hours to 1.8 hours [69].

Similar interventions were modelled to decrease the distance between a higher functioning facility that routinely conducts caesarean deliveries with the rest of the facilities in two regions of Ethiopia. Upgrading only seven selected health facilities improved the population served by first-level facilities located within the 2-hour transfer time to higher facilities from 70% to 80% (ie, translated into serving an additional 3.1 million people). Also, providing each facility with its transportation and communication if one or both were missing would result in 83% of the overall population being served by first-level facilities located within the 2-hour transfer time to higher facilities. Combining the above two interventions would improve the number of women and infants served by a first-level facility located within 2 hours of a facility providing lifesaving obstetric surgery from 70% to 90% (ie, translated into serving an additional 8.6 million populations) [64].

## DISCUSSION

To our knowledge, this is the first systematic review to map all studies that have applied geospatial analysis for maternal and neonatal morbidity and mortality in SSA. We found that geospatial analysis was commonly used to identify inequalities in maternal and neonatal morbidity, mortality, and health care utilization and identify gaps in the availability and geographic accessibility of maternal health facilities. In addition, we found few studies that used geospatial analysis for modelling intervention areas.

Of the several applications of geospatial analysis, the most used application was the identification of areas with significantly higher and lower maternal and neonatal morbidity, mortality, and health care utilization (such as antenatal care, skilled birth attendance, and postnatal care). The majority of the studies reported significant disparities in maternal and neonatal morbidity, mortality, and health care utilization at regional, national, or subnational levels. For instance, in Ethiopia, maternal and neonatal morbidity, mortality, and health care utilization were better in urban and semi-urban areas such as Addis Ababa, Dire Dawa, Harari, and Tigray; whereas, in Nigeria and Ghana, most studies reported a significant north-south divide. The findings of those studies could be used as input for prioritizing certain areas for resource allocation during national health planning. In addition, some studies pinpointed small areas sub-nationally with disproportional morbidity, mortality, and health care utilization which can be valuable input for subnational or local health planning according to the extent of the study. However, the availability of reliable, up-to-date, small-scale georeferenced data are still limited. This might require primary georeferenced data collection, which can be costly. Most of the reviewed studies use DHS as a data source. The DHS Program is United States Agency for International Development (USAID) funded nationally representative survey on fertility, family planning, maternal and child health, gender, HIV/AIDS, malaria, and nutrition for over 90 developing countries [70]. The finding that most of the reviewed studies use DHS as a data source is comparable with the finding of other reviews conducted on different study populations [71] and study areas [72]. Despite the large sample size and availability in regular five-year intervals, DHS only contains data aggregated at the survey cluster level [70]. Availability of household or individual level georeferenced data could further enable a more accurate prediction of the inequality with further resolution. In addition, most of the reviewed studies were done in a few countries, leaving parts of SSA not covered at all or only investigated as part of a few regional studies. All the national and subnational geospatial studies on maternal and neonatal health were done in 11 out of the 49 SSA countries. More than a quarter of those national and subnational studies were conducted in one country, Ethiopia. On the other hand, the number of geospatial studies done after 2010 is more than five times the number of studies done between 2000 and 2009, indicating better progress recently.

The other identified application of geospatial studies in maternal and neonatal health was the identification of gaps in maternal health care availability and geographic accessibility. We saw that though overall, women and neonates' geographic access to and availability of health facilities in SSA is low, this varies significantly between different countries in the region and between areas within countries. We also observed how the national summary estimates of availability and geographic accessibility, which are usually the ones used for monitoring purposes by countries, may obscure marked subnational variation. Such findings have two major implications. First, it illustrates that using national summary estimates of availability and geographic accessibility singlehandedly for monitoring purposes could be misleading. Hence, national-level availability indicators, such as the WHO's “at least one is C-EmONC for every 500 000 populations” indicator [73] and geographical accessibility targets such as Global Surgery's “women should be able to reach a lifesaving blood transfusion or caesarean sections within a maximum of two hours of travel” indicator [74], should always be used jointly with the performance of similar indicators sub-nationally. Second, such a finding also indicates which areas in the country are performing better than others regarding the availability and geographic accessibility of maternal health facilities. Such knowledge is essential for planning where to focus scarce resources for improving the availability and geographic accessibility of maternal health facilities. However, we also identified several limitations on geospatial studies on maternal facilities' availability and geographic accessibility. First, most geospatial models used to estimate the travel time to a health facility assumes patients to walk to the nearest road and then continue the journey driving. Yet, walking is the predominant form of transportation in rural Africa due to the lack of infrastructure and motorized transport services [75]. Indeed, a study in Siaya County of Kenya showed that 53 to 87 percent of women walked to the nearest maternal health care facility [61,76]. In addition, the travel speed assumptions used by geospatial models for mechanized transport were generous. For instance, for most studies, travel time was calculated by assuming a driving speed of 100, 50, and 30 km/h for primary, secondary, and tertiary roads, respectively, while a walking speed of 5 km/h was set to areas without a road. Studies conducted in Rwanda [77] and Sierra Leone [78] checked the agreement between travel time reported by the patient and calculated by the geospatial model with the above travel speed assumptions. In both studies, patient-reported travel time was longer than estimated by the geospatial model. As a result, those estimations usually underrate the actual time it takes a patient to reach a health facility. However, when conservative estimates of 50 km/h to primary roads, 20 km/h to secondary roads, and 5 km/h to tertiary roads while walking speed of 1.5-2.5 km/h for all other areas were used, the travel time estimated by the geospatial model was closer to the patient-reported time [78]. Moreover, most geospatial studies ignored seasonal variation when estimating geographic access to health facilities. The speed assumed for studies applies to the dry season. Yet, spatial access to health facilities generally decreases during the wet season for all modes of transport due to the increase in travel times imposed by precipitation and flooding. For instance, in southern Mozambique, at the peak of the dry season, nearly half of women lived within a two-hour travel time to the nearest C-EmONC facility that offers lifesaving maternal care. The population of women dropped twenty-seven percent during the rainy season because of weather-induced increases in travel in travel time. What is more, thirteen of the 417 neighbourhoods were isolated entirely from health facilities at some point [57]. Furthermore, studies about the accessibility of inter-facility referrals were usually estimations of the direct travel times, which did not consider the availability of transportation and communication delays in facilities. For instance, in a remote region in western Tanzania, the estimated median direct travel times between pairs of a facility with and without a caesarean section (ie, not considering the availability of transportation and communication delays) was around 26 minutes. However, less than two percent of the referring facilities had an ambulance at the facility. To access the remaining facilities, the patient has to either find a means of transportation herself (53%) or has to wait for an ambulance to come from other facilities such as the district health office (45%) [79]. Also, 75% do not have a mobile phone, a landline (phone/radio receiver), or both. Therefore, median direct travel times between pairs of facilities with and without caesarean section becomes 107 minutes [79].

Modelling and predictions based on the existing spatial data are one application of geospatial techniques. However, our review revealed that this application was not commonly used in SSA. The few studies in this regard quantified the potential effect of new interventions such as improving interfaculty transportation and communication, and upgrading strategically selected facilities to enhance access to health facilities [64,69]. In addition to quantifying the potential effect of an intervention on a population, modelling and prediction geospatial studies can be used to indicate what percentage of the population would benefit from a specific intervention, such as building a new health facility or road construction in a specific area. It can also indicate geographic areas that would benefit most from a selected intervention. Such application of geospatial techniques is specifically vital in resource-limited areas like SSA to help policymakers, and health planners make evidence-based decisions. However, more geospatial modelling studies are needed to learn about the specific advantages and limitations of the method.

This review has several strengths and limitations. It is the first study to review the application of geospatial techniques in maternal and neonatal health in SSA. Additionally, in consultation with a medical information specialist, we developed a broader search strategy to minimize the chance of missing relevant studies. Hence, we screened a total of 8184 articles for eligibility. Furthermore, there was no language or time restriction in the inclusion of articles. However, our study also has limitations that should be considered during the interpretation of the results. First, studies included in this review used different geospatial analysis methods; hence they are not exactly comparable to each other. Additionally, we only included articles indexed in PubMed, SCOPUS, EMBASE, and Web of Science databases; hence, there is a possibility that some potentially relevant studies indexed elsewhere may have been missed. We could not manage two databases we planned to search, which were African Journals Online (AJOL) and WHO Global Health Library. WHO Global Health Library was not accessible, while AJOL was difficult to search using Boolean operators. Finally, we could not do a meta-analysis due to the heterogeneity of the studies.

In conclusion, the use of geospatial analysis for maternal and neonatal health in SSA is still limited, and more detailed spatial data are required to exploit the potential of geospatial technologies fully. The available studies used geospatial analysis to identify inequalities in maternal and neonatal morbidity, mortality, and health care utilization and identify gaps in maternal health facilities' availability and geographic accessibility. In addition, we identified a few studies that used geospatial analysis for modelling intervention areas. The findings of those studies could be used as input for planning where to focus the scarce resources by prioritizing areas where maternal and neonatal morbidity and mortality are most likely to occur. On the other hand, we also identified challenges and shortcomings of the reviewed studies. First, the availability of reliable, up-to-date, small-scale georeferenced data are still a challenge. Similarly, women's access to health facilities should be perceived as more complex than the availability of a health facility and the calculated distance to a health facility.

## Additional material


Online Supplementary Document
