# Supplementary material for: How applicable is geospatial analysis in maternal and neonatal health in sub-Saharan Africa? A systematic review
Source: J Glob Health. 2022 Aug 9;12:04066. doi: 10.7189/jogh.12.04066 (PMC9359463; doi:10.7189/jogh.12.04066)
Supplement: Online Supplementary Document [file jogh-12-04066-s001.pdf]

Appendix S1: PRISMA 2009 Checklist

Section/topic	#	Checklist item	Reported on page #
**TITLE**			
Title	1	Identify the report as a systematic review, meta-analysis, or both.	Page 1
**ABSTRACT**			
Structured summary	2	Provide a structured summary including, as applicable: background; objectives; data sources; study eligibility criteria, participants, and interventions; study appraisal and synthesis methods; results; limitations; conclusions and implications of key findings; systematic review registration number.	Page 1
**INTRODUCTION**			
Rationale	3	Describe the rationale for the review in the context of what is already known.	Page 2
Objectives	4	Provide an explicit statement of questions being addressed with reference to participants, interventions, comparisons, outcomes, and study design (PICOS).	Page 2
**METHODS**			
Protocol and registration	5	Indicate if a review protocol exists, if and where it can be accessed (e.g., Web address), and, if available, provide registration information including registration number.	Page 2
Eligibility criteria	6	Specify study characteristics (e.g., PICOS, length of follow-up) and report characteristics (e.g., years considered, language, publication status) used as criteria for eligibility, giving rationale.	Page 3
Information sources	7	Describe all information sources (e.g., databases with dates of coverage, contact with study authors to identify additional studies) in the search and date last searched.	Page 2-3
Search	8	Present full electronic search strategy for at least one database, including any limits used, such that it could be repeated.	Appendix S2
Study selection	9	State the process for selecting studies (i.e., screening, eligibility, included in systematic review, and, if applicable, included in the meta-analysis).	Page 3
Data collection process	10	Describe method of data extraction from reports (e.g., piloted forms, independently, in duplicate) and any processes for obtaining and confirming data from investigators.	Page 3
Data items	11	List and define all variables for which data were sought (e.g., PICOS, funding sources) and any assumptions and simplifications made.	Page 3
Risk of bias in individual studies	12	Describe methods used for assessing risk of bias of individual studies (including specification of whether this was done at the study or outcome level), and how this information is to be used in any data synthesis.	Page 3
Summary measures	13	State the principal summary measures (e.g., risk ratio, difference in means).	Page 3
Synthesis of results	14	Describe the methods of handling data and combining results of studies, if done, including measures of consistency (e.g., I^2) for each meta-analysis.	Page 3

Section/topic	#	Checklist item	Reported on page #
Risk of bias across studies	15	Specify any assessment of risk of bias that may affect the cumulative evidence (e.g., publication bias, selective reporting within studies).	N/A
Additional analyses	16	Describe methods of additional analyses (e.g., sensitivity or subgroup analyses, meta-regression), if done, indicating which were pre-specified.	N/A
**RESULTS**			
Study selection	17	Give numbers of studies screened, assessed for eligibility, and included in the review, with reasons for exclusions at each stage, ideally with a flow diagram.	Page 4
Study characteristics	18	For each study, present characteristics for which data were extracted (e.g., study size, PICOS, follow-up period) and provide the citations.	Page 4
Risk of bias within studies	19	Present data on risk of bias of each study and, if available, any outcome level assessment (see item 12).	N/A
Results of individual studies	20	For all outcomes considered (benefits or harms), present, for each study: (a) simple summary data for each intervention group (b) effect estimates and confidence intervals, ideally with a forest plot.	Page 4-7
Synthesis of results	21	Present results of each meta-analysis done, including confidence intervals and measures of consistency.	N/A
Risk of bias across studies	22	Present results of any assessment of risk of bias across studies (see Item 15).	N/A
Additional analysis	23	Give results of additional analyses, if done (e.g., sensitivity or subgroup analyses, meta-regression [see Item 16]).	N/A
**DISCUSSION**			
Summary of evidence	24	Summarize the main findings including the strength of evidence for each main outcome; consider their relevance to key groups (e.g., healthcare providers, users, and policy makers).	Page 7-9
Limitations	25	Discuss limitations at study and outcome level (e.g., risk of bias), and at review-level (e.g., incomplete retrieval of identified research, reporting bias).	Page 9
Conclusions	26	Provide a general interpretation of the results in the context of other evidence, and implications for future research.	Page 9
**FUNDING**			
Funding	27	Describe sources of funding for the systematic review and other support (e.g., supply of data); role of funders for the systematic review.	Page 9

Appendix S2: Search strategy

PubMed

Search	Keywords	Search string	Items found* (09/01/2021)
#1	Maternal	("Maternal Health"[Mesh] OR "Pregnancy"[Mesh] OR "Postpartum Period"[Mesh] OR "Pregnancy Complications"[Mesh] OR "Maternal Mortality"[Mesh] OR "Perinatal Care"[Mesh] OR "Prenatal Care"[Mesh] OR "Postnatal Care"[Mesh] OR pregnan*[tiab] OR matern*[tiab] OR prenatal*[tiab] OR antenatal*[tiab] OR perinatal*[tiab] OR postnatal*[tiab] OR Parturition*[tiab] OR Childbirth*[tiab] OR Labor*[tiab] OR Puerperi*[tiab] OR Postpart*[tiab])	1,916,176
#2	Neonatal	("Infant, Newborn"[Mesh] OR "Infant Health"[Mesh] OR "Perinatal Mortality"[Mesh] OR "Perinatal Death"[Mesh] OR Newborn*[tiab] OR “New born*”[tiab] OR Neonat*[tiab])	807,535
#3	Spatial	("Spatial Analysis"[Mesh] OR "Geographic Information Systems"[Mesh] OR Spatial*[tiab] OR Kriging*[tiab] OR Spatiotemporal*[tiab] OR "Spatio Temporal*"[tiab] OR Spatio-Temporal*[tiab] OR Georeferenc*[tiab] OR Mapping[tiab] OR "Geographic Information System*" [tiab] OR GIS[tiab] OR "Hotspot analys*"[tiab] OR geospatial*[tiab] OR "Cluster analys*"[tiab])	588,052
#4	Sub-Saharan Africa	("Africa South of the Sahara"[Mesh] OR Sub-Sahara*[tiab] OR Subsahara*[tiab] OR "Central Africa*"[tiab] OR Cameroon*[tiab] OR "Central African Republic"[tiab] OR Chad*[tiab] OR Congo*[tiab] OR "Equatorial Guinea*"[tiab] OR Equatoguinean*[tiab] OR Gabon*[tiab] OR "Sao Tome and Principe"[tiab] OR "Sao Tomean*"[tiab] OR "Eastern Africa*"[tiab] OR "East Africa*"[tiab] OR Burundi*[tiab] OR Djibouti*[tiab] OR Eritrea*[tiab] OR Ethiopia*[tiab] OR Kenya*[tiab] OR Rwand*[tiab] OR Somali*[tiab] OR Sudan*[tiab] OR Tanzania*[tiab] OR Zanzibar*[tiab] OR Uganda*[tiab] OR "Southern Africa*"[tiab] OR Angol*[tiab] OR Botswan*[tiab] OR Eswatini[tiab] OR Lesotho*[tiab] OR Basotho[tiab] OR Malawi*[tiab] OR Mozambi*[tiab] OR Namibia*[tiab] OR "South Africa*"[tiab] OR Zambia*[tiab] OR Zimbabwe*[tiab] OR "Western Africa*"[tiab] OR "West Africa*"[tiab] OR Benin*[tiab] OR "Burkina Faso*"[tiab] OR Burkinabe*[tiab] OR "Cabo Verde*"[tiab] OR "Cape Verde*"[tiab] OR "Cote d`ivoire*"[tiab] OR "Ivory Coast*"[tiab] OR Ivorian*[tiab] OR Gambia*[tiab] OR Ghan*[tiab] OR Guinea*[tiab] OR Liberia*[tiab] OR Mali*[tiab] OR Mauritania*[tiab] OR Niger*[tiab] OR Nigeria*[tiab] OR Senegal*[tiab] OR "Sierra Leone*"[tiab] OR Togo*[tiab])	1,040,815
#5		("animals"[MeSH] NOT "humans"[MeSH])	4,775,702
#6		#1 OR #2	2,409,322

#7		#3 AND #4 AND #6	1,428
**#8**	**Final**	**#7 NOT #5**	**1,256**

EMBASE

Search		Query	Items found* (09/01/2021)
#1	Maternal	('obstetric procedure'/exp OR 'pregnancy disorder'/exp OR 'childbirth'/exp OR 'pregnancy'/exp OR 'puerperium'/exp OR 'maternal morbidity'/exp OR 'maternal mortality'/exp OR 'parental death'/exp OR (pregnan* OR matern* OR prenatal* OR antenatal* OR perinatal* OR postnatal* OR Parturition* OR Childbirth* OR Labor* OR Puerperi* OR Postpart*):ab,ti)	2,562,726
#2	Neonatal	('newborn'/exp OR 'newborn disease'/exp OR 'perinatal mortality'/exp OR 'perinatal morbidity'/exp OR 'newborn death'/exp OR (Newborn* OR 'New born*' OR Neonat*):ab,ti)	2,270,418
#3	Spatial	('spatial analysis'/exp OR 'geospatial analysis'/exp OR 'geographic information system'/exp OR 'geographic distribution'/exp OR (Spatial* OR Kriging* OR Spatiotemporal* OR 'Spatio Temporal*' OR Spatio-Temporal* OR Georeferenc* OR Mapping OR 'Geographic Information System*' OR GIS OR 'Hotspot analys*' OR geospatial* OR 'Cluster analys*'):ab,ti)	802,030
#4	Sub-Saharan Africa	('Africa south of the Sahara'/exp OR (Sub-Sahara* OR Subsahara* OR 'Central Africa*' OR Cameroon* OR 'Central African Republic' OR Chad* OR Congo* OR 'Equatorial Guinea*' OR Equatoguinean* OR Gabon* OR 'Sao Tome and Principe' OR 'Sao Tomean*' OR 'Eastern Africa*' OR 'East Africa*' OR Burundi* OR Djibouti* OR Eritrea* OR Ethiopia* OR Kenya* OR Rwand* OR Somali* OR Sudan* OR Tanzania* OR Zanzibar* OR Uganda* OR 'Southern Africa*' OR Angol* OR Botswan* OR Eswatini OR Lesotho* OR Basotho OR Malawi* OR Mozambi* OR Namibia* OR 'South Africa*' OR Zambia* OR Zimbabwe* OR 'Western Africa*' OR 'West Africa*' OR Benin* OR 'Burkina Faso*' OR Burkinabe* OR 'Cabo Verde*' OR 'Cape Verde*' OR 'Cote d'ivoire*' OR 'Ivory Coast*' OR Ivorian* OR Gambia* OR Ghan* OR Guinea* OR Liberia* OR Mali* OR Mauritania* OR Niger* OR Nigeria* OR Senegal* OR 'Sierra Leone*' OR Togo*):ab,ti)	1,360,137
#5		('animal'/exp NOT 'human'/exp)	5,549,780
#6		#1 OR #2	4,200,735
#7		#3 AND #4 AND #6	3,712
**#8**	**Final**	**#7 NOT #5**	**3,361**

Web of Science

Search		Query	Items found* (09/01/2021)
#1	Maternal	TS=(pregnan* OR matern* OR prenatal* OR antenatal* OR perinatal* OR postnatal* OR Parturition* OR Childbirth* OR Labor* OR Puerperi* OR Postpart*)	1,549,652
#2	Neonatal	TS=(Newborn* OR 'New born*' OR Neonat*)	317,002
#3	Spatial	TS=(Spatial* OR Kriging* OR Spatiotemporal* OR 'Spatio Temporal*' OR Spatio-Temporal* OR Georeferenc* OR Mapping OR 'Geographic Information System*' OR GIS OR 'Hotspot analys*' OR geospatial* OR 'Cluster analys*')	2,080,039
#4	Sub-Saharan Africa	TS= (Sub-Sahara* OR Subsahara* OR 'Central Africa*' OR Cameroon* OR 'Central African Republic' OR Chad* OR Congo* OR 'Equatorial Guinea*' OR Equatoguinean* OR Gabon* OR 'Sao Tome and Principe' OR 'Sao Tomean*' OR 'Eastern Africa*' OR 'East Africa*' OR Burundi* OR Djibouti* OR Eritrea* OR Ethiopia* OR Kenya* OR Rwand* OR Somali* OR Sudan* OR Tanzania* OR Zanzibar* OR Uganda* OR 'Southern Africa*' OR Angol* OR Botswan* OR Eswatini OR Lesotho* OR Basotho OR Malawi* OR Mozambi* OR Namibia* OR 'South Africa*' OR Zambia* OR Zimbabwe* OR 'Western Africa*' OR 'West Africa*' OR Benin* OR 'Burkina Faso*' OR Burkinabe* OR 'Cabo Verde*' OR 'Cape Verde*' OR 'Cote d'ivoire*' OR 'Ivory Coast*' OR Ivorian* OR Gambia* OR Ghan* OR Guinea* OR Liberia* OR Mali* OR Mauritania* OR Niger* OR Nigeria* OR Senegal* OR 'Sierra Leone*' OR Togo*)	1,088,591
#5		#1 OR #2	1,752,665
**#6**	**Final**	**#3 AND #4 AND #5**	**3,184**

SCOPUS

Search		Query	Items found* (05/01/2021)
#1	Maternal	TITLE-ABS-KEY(pregnan* OR matern* OR prenatal* OR antenatal* OR perinatal* OR postnatal* OR Parturition* OR Childbirth* OR Labor* OR Puerperi* OR Postpart*)	3,516,515
#2	Neonatal	TITLE-ABS-KEY(Newborn* OR "New born*" OR Neonat*)	995,538
#3	Spatial	TITLE-ABS-KEY(Spatial* OR Kriging* OR Spatiotemporal* OR "Spatio Temporal*" OR Spatio-Temporal* OR Georeferenc* OR Mapping OR "Geographic Information System*" OR GIS OR "Hotspot analys*" OR geospatial* OR "Cluster analys*")	2,729,366

#4	Sub-Saharan Africa	TITLE-ABS-KEY(Sub-Sahara* OR Subsahara* OR "Central Africa*" OR Cameroon* OR "Central African Republic" OR Chad* OR Congo* OR "Equatorial Guinea*" OR Equatoguinean* OR Gabon* OR "Sao Tome and Principe" OR "Sao Tomean*" OR "Eastern Africa*" OR "East Africa*" OR Burundi* OR Djibouti* OR Eritrea* OR Ethiopia* OR Kenya* OR Rwand* OR Somali* OR Sudan* OR Tanzania* OR Zanzibar* OR Uganda* OR "Southern Africa*" OR Angol* OR Botswan* OR Eswatini OR Lesotho* OR Basotho OR Malawi* OR Mozambi* OR Namibia* OR "South Africa*" OR Zambia* OR Zimbabwe* OR "Western Africa*" OR "West Africa*" OR Benin* OR "Burkina Faso*" OR Burkinabe* OR "Cabo Verde*" OR "Cape Verde*" OR "Cote d'ivoire*" OR "Ivory Coast*" OR Ivorian* OR Gambia* OR Ghan* OR Guinea* OR Liberia* OR Mali* OR Mauritania* OR Niger* OR Nigeria* OR Senegal* OR "Sierra Leone*" OR Togo*)	1,876,837
#5		#1 OR #2	4,117,575
**#6**	**Final**	**#3 AND #4 AND #5**	**3,698**

Supplementary file 3 - Characteristics of included studies

Author & Year of publication	Focus	Scope of the study	Country	Outcome	Study design	Data source	Study period	Spatial analysis method	Funding
Gayawan 2014	Maternal	National	Nigeria	SBA	Cross sectional study	DHS survey	2000 to 2009	Global spatial autocorrelation; Spatial interpolation; Hot spot analysis; Spatial scan statistics; Geographic Weighted Regression	Not mentioned
Gayawan 2014	Maternal	National	Nigeria	ANC	Cross sectional study	DHS survey	2000 to 2009	Global spatial autocorrelation; Incremental autocorrelation; Hot spot analysis; Spatial scan statistics; Spatial interpolation	Not mentioned
O'Meara 2013	Maternal	Sub-national	Kenya	ANC	Cross sectional study	Others	2010 to 2014	Global spatial autocorrelation; Spatial interpolation; Spatial scan statistics; Different logistic regression models	Not mentioned
Masters 2013	Maternal	Sub-national	Ghana	ANC & SBA	Cross sectional study	DHS survey; EmONC survey; Road network data; Others	2010 to 2014	Global spatial autocorrelation; Spatial interpolation; Spatial scan statistics; Different logistic regression models	Funded
Gething 2012	Maternal	National	Ghana	Access to health care	Cross sectional study	Others	2000 to 2009	Global spatial autocorrelation; Hot spot analysis; Spatial interpolation; Spatial scan statistics	Not mentioned
Gabrysch 2011	Maternal	National	Zambia	Access to health care	Cross sectional study	Health Facility Assessment	2000 to 2009	Global spatial autocorrelation; Hot spot analysis; Incremental spatial autocorrelation	Not mentioned
Bailey 2011	Both	Sub-national	Ethiopia	Access to health care	Cross sectional study	EmONC survey; LandScan population data; Road network data	2000 to 2009	Global spatial autocorrelation; Hot spot analysis; Incremental spatial autocorrelation	Funded
Kazembe 2010	Neonatal	National	Malawi	Neonatal mortality	Cross sectional study	DHS survey	2000 to 2009	Global spatial autocorrelation; Local spatial autocorrelation; parallel coordinate plot (PCP)	No funding
Adebayo 2004	Neonatal	National	Nigeria	Neonatal mortality	Cross sectional study	DHS survey	<2000	The Bernoulli model; Anselin Local Moran's I; Different logistic regression models	Not mentioned
Mwaliko 2014	Maternal	Sub-national	Kenya	SBA	Cross sectional study	Health and Demographic Surveillance System	2000 to 2009	Travel time/accessibility modeling; Hot spot analysis; Different logistic regression models	Not mentioned
Nesbitt 2014	Maternal	Sub-national	Ghana	SBA	Cross sectional study	Health and Demographic Surveillance System	2000 to 2009	Global spatial autocorrelation; Spatial scan statistics; Different logistic regression models	Funded
Agadjanian 2016	Maternal	Sub-national	Mozambique	SBA	Cohort study	Secondary analysis of cohort data	2000 to 2009	Linear regression; Exploratory spatial analysis; Local spatial autocorrelation	Funded
Banda 2016	Neonatal	Sub-national	Malawi	Perinatal Mortality	Cross sectional study	Secondary analysis of (randomized controlled) trial	2010 to 2014	Global spatial autocorrelation; Geographic Weighted Regression (GWR); Local spatial autocorrelation	Not mentioned

Sprague 2016	Maternal	National	Uganda	SBA	Cohort study	DHS survey; Primary data collection; Others	2010 to 2014	Exploratory spatial analysis; Different logistic regression models; Spatial scan statistics	Not mentioned
Banda 2016	Maternal	National	Zambia	Pregnancy-related mortality	Cross sectional study	Census	2010 to 2014	Linear regression; Spatial interpolation; Travel time/accessibility modeling	Funded
Bosomprah 2016	Maternal	National	Ghana	SBA	Cross sectional study	HIMS; Others	2010 to 2014	Spatial interaction model; Zero-inflated Poisson models; Zero-inflated negative binomial model	Not mentioned
Kayode 2017	Neonatal	Regional	Regional	Neonatal mortality	Other: ecological study	Others	2010 to 2014	Travel time/accessiblity modeling; Birth density by ward	Funded
Grady 2017	Neonatal	Regional	Regional	Neonatal mortality	Cross sectional study	DHS survey	2010 to 2014	Travel time/accessibility modeling; Bland-Altman analysis	Not mentioned
Yeneneh 2018	Maternal	National	Ethiopia	ANC	Cross sectional study	DHS survey	2010 to 2014	Exploratory spatial analysis; Buffer analysis	Not mentioned
Manyeh 2018	Maternal	Sub-national	Tanzania	Pregnancy-related mortality	Cross sectional study	Health and Demographic Surveillance System	2010 to 2014	Spatial quantile regression; Different bayesian models	Funded
Iyanda 2018	Maternal	National	Ghana	ANC & SBA	Cross sectional study	DHS survey	2010 to 2014	Zero-inflated negative binomial model; Different bayesian models	Not mentioned
Yao 2018	Maternal	Sub-national	Mozambique	Access to health care	Cohort study	Secondary analysis of cohort data	2010 to 2014	Spearman rank correlation; Different logistic regression models	Not mentioned
Tlou 2017	Maternal	Sub-national	South Africa	Pregnancy-related mortality	Cross sectional study	Secondary analysis of cohort data	2010 to 2014	Predicting priority area; Different logistic regression models	No funding
Ouko 2019	Maternal	Sub-national	Kenya	SBA	Cross sectional study	DHS survey; Primary data collection; Kenya Master Health Facility Listing	2015 - 2021	Exploratory spatial analysis; Different logistic regression models	Not mentioned
Adedokun 2019	Maternal	National	Nigeria	SBA	Cross sectional study	DHS survey	2010 to 2014	Spatial scan statistics; Different logistic regression models	Funded
Gao 2019	Maternal	National	Kenya	Access to health care	Cross sectional study	DHS survey	2010 to 2014	Different Bayesian models; Exploratory spatial analysis	No funding
Sisay 2019	Maternal	National	Ethiopia	PNC	Cross sectional study	DHS survey	2015 - 2021	Global spatial autocorrelation; Exploratory spatial analysis	No funding
Keyes 2019	Both	National	Mozambique	Access to health care	Cross sectional study	EmONC survey	2010 to 2014	Different Bayesian models; Geoadditive multi-categorical response model (state-specific spatial effects)	Not mentioned
Nigatu 2019	Maternal	Sub-national	Ethiopia	SBA	Cross sectional study	Primary data collection	2015 - 2021	Multivariate probit model; Hot spot analysis	Funded
Owoo 2019	Maternal	National	Ghana	Abortion	Cross sectional study	Other: 2017 Ghana Maternal Health Survey	2015 - 2021	Spatial regression; Hot spot analysis	No funding

Kohler 2019	Maternal	Sub-national	Kenya	ANC	Cross sectional study	Others	2010 to 2014	Global spatial autocorrelation; Hot spot analysis	Not mentioned
Tegegne 2019	Maternal	National	Ethiopia	ANC	Cross sectional study	DHS survey	2015 - 2021	Global spatial autocorrelation; Hot spot analysis	No funding
Defar 2019	Both	Sub-national	Ethiopia	ANC	Cross sectional study	Secondary analysis of (randomized controlled) trial	2015 - 2021	Different logistic regression models; intrinsic conditional autoregressive prior	Funded
Ononokpono 2020	Maternal	Regional	Regional	Access to health care	Cross sectional study	Census; WorldPop database	2015 - 2021	Spatial scan statistics; Spatial interpolation	Funded
Muluneh 2020	Maternal	National	Ethiopia	SBA	Case control study	DHS survey	2015 - 2021	Global spatial autocorrelation; Spatial scan statistics	No funding
Tessema 2020	Maternal	National	Ethiopia	ANC	Cross sectional study	DHS survey	2015 - 2021	Anselin Local Moran's I; Spatial scan statistics	No funding
Ononokpono 2020	Maternal	National	Nigeria	PNC	Cross sectional study	DHS survey	2010 to 2014	Global spatial autocorrelation; Spatial scan statistics	Not mentioned
Tesema 2020	Maternal	National	Ethiopia	Abortion	Cross sectional study	DHS survey	2015 - 2021	Linear regression; Travel time/accessibility modeling	No funding
Tessema 2020	Maternal	National	Ethiopia	SBA	Cross sectional study	DHS survey	2015 - 2021	Linear regression; Travel time/accessibility modeling	Not mentioned
Schmitz 2020	Both	Sub-national	Tanzania	Access to health care	Cross sectional study	Health Facility Assessment	2015 - 2021	Poisson regression model with geo-additive predictors	Funded
Dotse-Gborgbortsi 2020	Maternal	Sub-national	Ghana	SBA	Cross sectional study	HIMS	2015 - 2021	Travel time/accessiblity modeling	Not mentioned
Kurji 2020	Maternal	Sub-national	Ethiopia	ANC, SBA & PNC	Cross sectional study	Secondary analysis of (randomised controlled) trial	2015 - 2021	Travel time/accessiblity modeling	Funded
Dotse-Gborgbortsi 2020	Maternal	National	Ghana	SBA	Cross sectional study	DHS survey; WorldPop database; HMIS; SPA	2015 - 2021	Travel time/accessiblity modeling	Funded
Wigley 2020	Maternal	Regional	Regional	Access to health care	Cross sectional study	Harvard Dataverse; WorldPop database	2015 - 2021	Different bayesian models	Not mentioned
Teshale 2020	Maternal	National	Ethiopia	SBA	Cross sectional study	DHS survey	2015 - 2021	Different bayesian models	No funding
Tesema 2020	Neonatal	National	Ethiopia	Stillbirth	Cross sectional study	DHS survey	2015 - 2021	Global spatial autocorrelation	No funding
Tegegne 2020	Maternal	National	Ethiopia	SBA	Cross sectional study	DHS survey; SPA	2015 - 2021	Spatial scan statistics	No funding
Tesema 2020	Maternal	National	Ethiopia	SBA	Cross sectional study	DHS survey	2015 - 2021	Spatial scan statistics	No funding
Yadeta 2020	Neonatal	National	Ethiopia	Perinatal Mortality	Cross sectional study	DHS survey	2015 - 2021	Travel time/accessiblity modeling	No funding

Tessema 2020	Maternal	National	Ethiopia	ANC	Cross sectional study	DHS survey	2015 - 2021	Travel time/accessiblity modeling	Not mentioned
Dankwah 2021	Maternal	National	Ghana	PNC	Cross sectional study	DHS survey	2010 to 2014	Different Bayesian models	No funding
TatendaMakanga 2017	Maternal	Sub-national	Mozambique	Access to health care	Cross sectional study	Secondary analysis of (randomised controlled) trial	2010 to 2014	Travel time/accessiblity modeling	Funded
Wong 2018	Maternal	National	Nigeria	SBA	Cross sectional study	DHS survey	2010 to 2014	Travel time/accessiblity modeling	Funded
Gayawan 2016	Maternal	Regional	Regional	ANC	Cross sectional study	DHS survey	2010 to 2014	Spatial scan statistics	Not mentioned
Chen 2017	Both	Sub-national	Tanzania	Access to health care	Cross sectional study	Health Facility Assessment; OpenStreetMap; Pregnancy Outcomes Mortality Surveillance system; Shuttle Radar Topography Mission digital elevation model data	2015 - 2021	Travel time/accessiblity modeling	Funded
Cavallaro 2020	Maternal	National	Senegal	Access to health care	Cross sectional study	DHS survey; SPA; Census	2015 - 2021	Travel time/accessiblity modeling	Funded

EmONC = Emergency Obstetric and Newborn Care, HIMS = Health Information Management System, SPA = Service Provision Assessment, DHS = Demographic and Health Survey, PNC = Postnatal Care, SBA = Skilled Birth Attendance, ANC = Antenatal Care
